# Expansive hematoma in delayed cerebral radiation necrosis in patients treated with T-DM1: a report of two cases

**DOI:** 10.1186/s12885-016-2464-1

**Published:** 2016-07-04

**Authors:** Koichi Mitsuya, Junichiro Watanabe, Yoko Nakasu, Nakamasa Hayashi, Hideyuki Harada, Ichiro Ito

**Affiliations:** Division of Neurosurgery, Shizuoka Cancer Center, 1007, Shimo-Nagakubo, Naga-izumi, Sunto, Shizuoka 411-8777 Japan; Division of Breast Oncology, Shizuoka Cancer Center, 1007, Shimo-Nagakubo, Naga-izumi, Sunto, Shizuoka 411-8777 Japan; Division of Radiation Oncology, Shizuoka Cancer Center, 1007, Shimo-Nagakubo, Naga-izumi, Sunto, Shizuoka 411-8777 Japan; Division of Pathology, Shizuoka Cancer Center, 1007, Shimo-Nagakubo, Naga-izumi, Sunto, Shizuoka 411-8777 Japan

**Keywords:** Brain metastasis, Breast cancer, Human epidermal growth factor receptor type 2, Radiation necrosis, Stereotactic radiosurgery, Trastuzumab emtansine

## Abstract

**Background:**

Multiple new targeted agents have been developed for patients with human epidermal growth factor receptor type 2 (HER2) – positive breast cancer. Patients with HER2– positive breast cancer will develop brain metastases with greater incidence than patients with non-HER2 cancers, and many of them will undergo stereotactic radiosurgery (SRS) or other CNS radiotherapy. The interaction between radiation effects and new targeted agents is not well understood. We report two cases suggesting a novel adverse effect of T-DM1 (trastuzumab emtansine) on symptomatic enlargement of radiation necrosis (RN) after SRS.

**Case presentation:**

Two patients with HER2-positive breast cancer had received SRS for single brain metastasis more than 5-years ago. They had been heavily treated for HER2-positive metastatic breast cancer (trastuzumab and pacritaxel, lapatinib and capecitabine). They initiated T-DM1 therapy for progressive systematic disease 5.5 years after stereotactic irradiation, when a small RN was recognized on brain MR images of each patient. The RN lesions increased in size and became symptomatic during 13 or 14 months of T-DM1 treatment. The patients underwent surgical resection of the lesion. Pathological examination revealed necrosis, hematoma, granulation tissue and telangiectasia without neoplastic cells.

**Conclusions:**

A potential enhancement of RN by T-DM1 in the brain may be one of important adverse events associated with the use of T-DM1 for patients after SRS. These cases highlight the need of careful follow-up when combining new systemic targeted therapies and SRS for brain metastases.

## Background

T-DM1 (Trastuzumab emtansine) is an antibody–drug conjugate combining the human epidermal growth factor receptor type 2 (HER2) - targeted monoclonal antibody, trastuzumab, with the microtubule inhibitor mertansine (DM1). Through the combined effect of inhibition of HER2 signaling and delivery of a cytotoxic agent to HER2 expressing cells, T-DM1 demonstrated improved progression-free and overall survival in patients with HER2-positive metastatic breast cancer. The most frequently reported adverse events of T-DM1 include fatigue, nausea, diarrhea, elevated transaminases, anemia, thrombocytopenia, and hemorrhage [1, 2]. Increased brain edema has been reported shortly after T-DM1 infusion in patients who received stereotactic radiosurgery (SRS) at a median of 8.5 days prior to the infusion, with potential interactions between T-DM1 and SRS [3]. Herein, we present two cases of histologically proven delayed cerebral radiation necrosis with rapid symptomatic growing mass sign associated with T-DM1 treatment, and describe possible mechanisms of its neurological toxicity.

## Case presentation

### Case 1

**Fig. 1:**
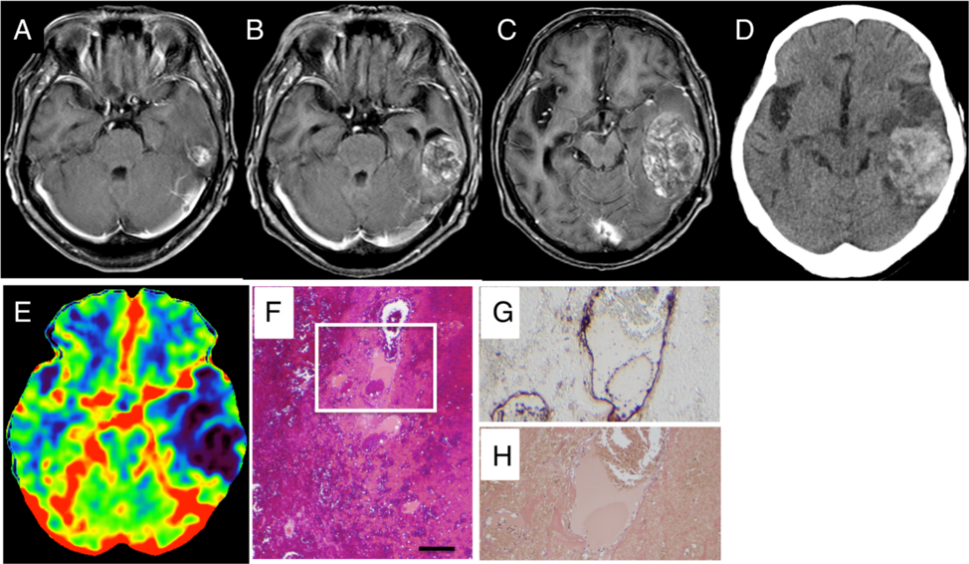
Axial contrast-enhanced MRI brain images showing a small delayed radiation necrosis in the area of a metastatic brain tumor treated by SRS (**a**) at initiation of T-DM1 (15 mm), (**b**) heterogeneous enhancement (42 mm) at 14 months and (**c**) rapid progression of nodular lesion (58 mm) at 15 months after initiation of T-DM1. Precontrast CT scan shows heterogeneous high density area in this lesion (**d**). The lesion is shown as an area of low cerebral blood volume (CBV) by perfusion CT (**e**). Photomicrographs of the removed lesion on Hematoxylin and Eosin (H-E) (**f**), CD31 (**g**) and Elastica van Gieson (EVG) (**h**) immunohistochemical stains. The scale bar represents 500 μm for panel. Hemorrhage and dilated vessels are shown (**f**). The area surrounded by *white square* is focused in f and g. CD31 immunostaining demonstrates endothelial cells surrounding dilated vascular lumina (**g**). However, they are not accompanied by perivascular structure, which are demonstrated as lacks of both *black* (corresponding to elastic fiber) and *dark brown lines* (corresponding to collagen fiber) in EVG stain (**h**)

A 67-year-old woman presented with a-week history of progressive sensory aphasia. Magnetic resonance imaging (MRI) of the brain showed a heterogeneous mass lesion in the left temporal lobe, which was increasing in size (Fig. 1a–c). Perfusion computed tomography (CT) of the head showed a decrease in cerebral blood volume (CBV) in the mass lesion (Fig. 1d).

Eight years earlier, the patient had presented with an inflammatory breast cancer of the left with estrogen receptor (ER) negative (0 %), progesterone receptor negative (0 %), HER2 positive (3+) and Ki67 unavailable. T4dN2M1 (HEP), stage IV and tumor size was 7 cm × 7.5 cm at initial diagnosis. She underwent the first-line chemotherapy as a palliation. She received 13 cycles of weekly paclitaxel with trastuzumab. At the time of disease progression, a screening brain MRI disclosed an asymptomatic brain metastasis (BM), 8 mm in diameter, in the left temporal lobe, and she underwent SRS (D95 = 25 Gy) for the BM. She received vinorelbine with trastuzumab, lapatinib with capecitabine, and weekly trastuzumab as palliative regimens. After 3 years from initial diagnosis, she underwent left mastectomy and was followed by radiotherapy against the chest wall with the aim of local control. Trastuzumab monotherapy was resumed as a systemic therapy, however, mediastinal lymph node and adrenal metastases were disclosed. She received lapatinib plus capecitabine therapy as treatment for the progression.

Approximately 6.5 years after initial diagnosis and 5.5 years after SRS, the patient received T-DM1, as a newly approved targeted agent. She received 3.6 mg per kilogram of body weight intra venously every 21 days. Her systemic metastases were then well controlled. Eight months after the initiation of T-DM1, a follow-up MRI revealed an asymptomatic cyst and nodular lesion at the irradiation site, which increased in size consistently (Fig. 1b). Cytological examination of the cyst fluid was negative for neoplastic cells. The images and cytological results were consistent with symptomatic RN. The patient underwent surgical resection of the mass lesion 14 months after the initiation of T-DM1. The pathological examination demonstrated peri-lesional gliosis and fibrinoid necrosis of vascular walls, which were the same findings as observed in usual post-radiation necrosis. No malignant cells were observed. Moreover, there were dilated vascular structures composed of only endothelial cells without collagen or elastic fiber lining in the hemorrhagic lesions (Fig. 1f to h). She gradually improved in sensory aphasia after surgery.

### Case 2

**Fig. 2:**
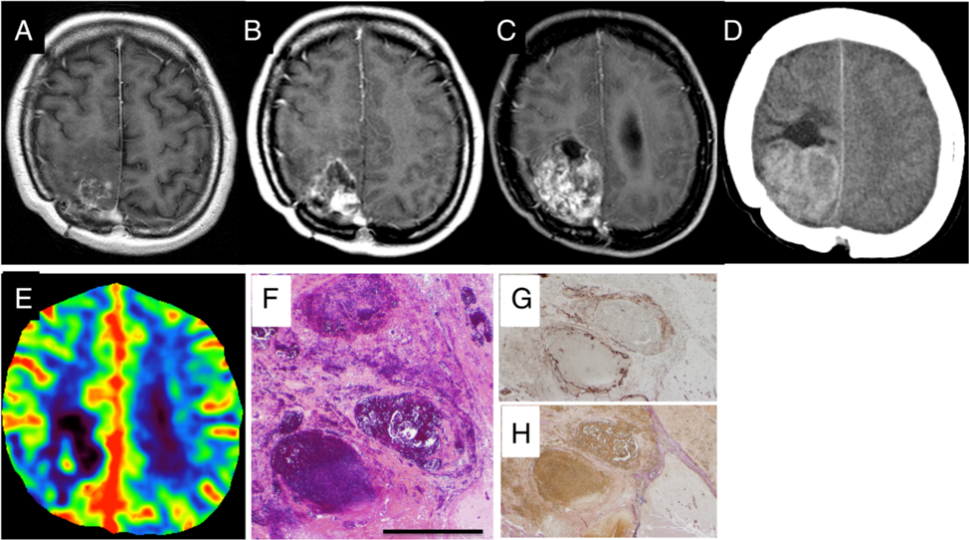
Axial contrast-enhanced MRI brain images showing a right parietal delayed radiation necrosis (15 mm) after SRS, surgery, and SRT at initiation of T-DM1 (**a**), (**b**) enlarging heterogeneous enhancement (30 mm) at 12 months (**c**) rapid progression of nodular lesion (48 mm) at 15 months after initiation of T-DM1 therapy. Precontrast CT scan shows high density area in this lesion (**d**). Perfusion CT (**e**) showing an area of low CBV. Photomicrographs of H-E (**f**), CD31 (**g**) and EVG (**h**) stains. The scale bar represents 500 μm for panel. Hemorrhage and dilated blood containing spaces are shown (**f**). CD31 immunostaining (**g**) shows vascular structures of variable sizes. However, EVG stain (**g**) indicates no elastic or collagenous fibers surrounding vascular lumina, in contrast to *purplish-brown* collagen and *black* elastic fibers comprising septa between hemorrhagic area (*left*) and viable cerebral tissue (*lower right*)

A 45-year-old woman presented with slight disorientation during follow-up for a brain metastasis treated with SRS, salvage removal, and salvage stereotactic radiotherapy. She had received T-DM1 with the aim of palliative systemic therapy for 15 months. Brain MRI demonstrated a rapidly enlarging mass lesion of heterogeneous enhancement with cysts in the right parietal lobe (Fig. 2a-c). Perfusion CT showed a decrease in CBV in the solid lesion (Fig. 2d).

Thirteen years earlier, the patient was referred to our hospital as having metastatic breast cancer with 2.5-years of disease-free interval from initial breast surgery. Her breast cancer was invasive ductal carcinoma, T2N1M0, stage IIB and tumor size was 3 cm × 3.5 cm at initial surgery. The systemic survey disclosed she was having systemic lymph node metastases. Biopsy specimen from the lymph node revealed luminal-HER2 feature; ER positive (50 %), PR positive (70 %), HER2 positive (3+) and Ki67 unavailable. She received 10 cycles of weekly paclitaxel with trastuzumab as first-line chemotherapy for metastatic disease, and then was maintained by trastuzumab and leuprorelin acetate with tamoxifen for 2 years. A check-up MRI revealed an asymptomatic brain metastasis, about 10 mm in diameter, in the right parietal lobe. She underwent SRS (D95 = 25 Gy). She also received trastuzumab with capecitabine for newly developed liver metastases for 4 months. A year later, a brain MRI demonstrated asymptomatic recurrence of the parietal metastasis. She underwent surgical removal and postoperative stereotactic radiotherapy (SRT) (30 Gy/5 fr) for the lesion because of tumor invasion into the arachnoid membrane. The recurrent metastasis had a pathological profile of ER negative, PR-negative and HER2 positive. She received weekly trastuzumab therapy, and added lapatinib for newly developed ovarian metastasis.

Approximately 14 years after initial diagnosis and 5.5 years after SRT, she received T-DM1. She received 3.6 mg per kilogram of body weight intra venously every 21 days. Her liver and ovarian metastases were then well controlled. Nine months after the initiation of T-DM1, a follow-up MRI revealed an asymptomatic cyst and nodular lesion at the irradiation site (Fig. 2a b), which increased in size consistently in 6 months (Fig. 2c). The features of imaging (Fig. 2d) and history of repeated stereotactic irradiation were consistent with the diagnosis of RN. She underwent surgical resection of the lesion within the safety margin. The pathological examination demonstrated an organizing hematoma without malignancy. The hematoma was surrounded by fibrous septa existing in viable cerebral tissue, and contained dilated vascular structures of various sizes, part of which looked conglomerated. There were no collagenous or elastic fiber components surrounding dilated CD31-positive endothelial lumina (Fig. 2f to h). She improved slight disorientation after surgery.

## Discussion

Delayed RN has been one of the critical problems for long-term survivors after SRS for brain metastases [4]. A report by the Radiation Therapy Oncology Group trial 90-05 involved a study of 156 patients with recurrent primary brain tumors or brain metastases [5]. From this trial, the group made SRS dose recommendations based on maximum tumor diameter to limit RN risk. In fact, dose-volume analysis of complication risk estimates were reported after SRS for arterio-venous malformations (AVM) [6, 7]. Recently, RN with “enhanced nodular lesion and cyst” was reported as a subtype of RN which develops more than 5 years after SRS for AVM [8, 9]. It consisted of a rapidly growing nodular lesion and hematoma, or cysts, causing neurological manifestations.

Here we report two cases of growing nodule plus cyst, which presented 6 and 8 years after SRS. For both patients, the nodules plus cysts were associated with treatment of stage IV breast cancer using T-DM1. Each lesion was identified as a small enhanced mass on MRI soon after commencement of T-DM1. Neurological deterioration, due to rapid growth of the lesion, occurred in both cases 13 and 14 months later. Pathologically, the lesions represented a mixture of granulation tissue, necrosis and hemorrhage, with telangiectasia and fibrinoid degeneration of the small vessels. The triggering role of T-DM1 in the induction of these lesions is supported by a chronological relationship between lesion development and T-DM1 exposure. Similar cases of cutaneous, or mucosal telangiectasia and hemorrhage have been reported in association with T-DM1 [10].

T-DM1 is an antibody-drug conjugate incorporating the HER2-targeted antitumor properties of trastuzumab with the cytotoxic activity of the microtubule-inhibitory agent DM1. T-DM1 is approved in several countries as a single agent for HER2 positive metastatic breast cancer (MBC), which has previously been treated with trastuzumab and a taxane. In a phase III EMILIA trial, T-DM1 significantly prolonged median progression-free survival (PFS; hazard ratio (HR) = 0.65; *P* < 0.001; 9.6 versus 6.4 months) and median overall survival (OS; HR = 0.68; *P* < 0.001; 30.9 versus 25.1 months) with less toxicity, compared with lapatinib with capecitabine in patients with HER2-positive MBC who previously treated with trastuzumab-based regimen [1]. Several reports have been published that indicated T-DM1 is also effective for brain metastasis [11–14]. The mild to moderate, bur continuous thrombocytopenia (28.0 %) is a common side effect of T-DM1.

Although the pathophysiology of the growing delayed RN is not fully understood, several mechanisms are hypothesized. First, an irradiated lesion makes nodular granulation with cysts slowly over a long period of time. In addition, the nodule contains abundant neo-vasculature that can easily cause micro-bleeding into the cysts, and from that, they can become expansive hematoma with mass effects [15]. Second, induced telangiectasias by T-DM1 may represent an additional cause of hemorrhage and growth of the lesion after SRS [16]. Third, thrombocytopenia by T-DM1 may also enhance hemorrhage from abnormal vessels in the lesion, although thrombocytopenia was not found in our patients from their T-DM1 treatment.

Furuse et al. reviewed pathologic features of radiation necrosis from 18 surgically treated patients. They found necrotic core and enlarged vessels with thin walls, hyalinized vessels and reactive astrocytes in surrounding area [17]. No massive hematomas were reported, though telangiectatic vessels were accompanied by microbleeding and interstitial edema. In both hemorrhagic lesions of our cases, there were dilated vascular structures composed of endothelial lining without fibrous components. However, it remains to be explored whether these thin-walled vessels would induce hemorrhage or would have been induced by the certain causes same as those of hemorrhage.

Before the introduction of trastuzumab, patients with HER2-positive MBC had very poor prognosis. As such, adverse events that affected the brain after SRS for brain metastases were never a problem that needed consideration. However, new targeted agents for HER2-positive MBC have significantly improved patient long-term survival. A novel association between T-DM1 and RN in the brain may be an important factor in the micro-bleeding adverse events or the mass effect associated with the use of T-DM1 for patients. These cases highlight the need to caution when combining radiation therapy for brain metastases and new systemic targeted therapies. Our observation suggests a need for a prospective evaluation for brain metastases in patients treated with T-DM1 after SRS.

## Conclusions

While this report is limited in two cases, the correlation of growing mass lesion in the area of SRS and T-DM1 treatment was salient. New targeted agents are introduced to clinical use every three weeks, providing new benefits for patients. SRS is used with increased frequency for small brain metastases, sometimes for asymptomatic ones. Thorough reporting and evaluation of adverse effects are essential in the concurrent use of new modalities.

## Abbreviations

AVM, arteriovenous malformation; BM, brain metastasis; CBV, cerebral blood volume; CT, computed tomography; DM1, mertansine; ER, estrogen receptor; HER2, human epidermal growth factor receptor type 2; HR, hazard ratio; MBC, metastatic breast cancer; MRI, magnetic resonance imaging; OS, overall survival; PFS, progression free survival; PR, progesterone receptor; RN, radiation necrosis; SRS, stereotactic radiosurgery; SRT, stereotactic radiotherapy; T-DM1, trastuzumab emtansine
